# Cornification of keratinocytes is associated with differential changes in the catalytic activity and the immunoreactivity of transglutaminase-1

**DOI:** 10.1038/s41598-023-48856-1

**Published:** 2023-12-06

**Authors:** Marta Surbek, Tessa Van de Steene, Attila Placido Sachslehner, Bahar Golabi, Johannes Griss, Sven Eyckerman, Kris Gevaert, Leopold Eckhart

**Affiliations:** 1 Department of Dermatology, Medical University of Vienna, Vienna, Austria; 2 VIB Center for Medical Biotechnology Center, VIB, Ghent University, Ghent, Belgium; 3 Department of Biomolecular Medicine, Ghent University, Ghent, Belgium

**Keywords:** Enzymes, Immunochemistry, Cell death, Skin diseases

## Abstract

Transglutaminase 1 (TGM1) plays an essential role in skin barrier formation by cross-linking proteins in differentiated keratinocytes. Here, we established a protocol for the antibody-dependent detection of TGM1 protein and the parallel detection of TGM activity. TGM1 immunoreactivity initially increased and co-localized with membrane-associated TGM activity during keratinocyte differentiation. TGM activity persisted upon further differentiation of keratinocytes, whereas TGM1 immunoreactivity was lost under standard assay conditions. Pretreatment of tissue sections with the proteases trypsin or proteinase K enabled immunodetection of TGM1 in cornified keratinocytes, indicating that removal of other proteins was a prerequisite for TGM1 immunolabeling after cornification. The increase of TGM activity and subsequent loss of TGM1 immunoreactivity could be replicated in HEK293T cells transfected with TGM1, suggesting that protein cross-linking mediated by TGM1 itself may lead to reduced recognition of TGM1 by antibodies. To screen for proteins potentially regulating TGM1, we performed Virotrap experiments and identified the CAPNS1 subunit of calpain as an interaction partner of TGM1. Treatment of keratinocytes and TGM1-transfected HEK293T cells with chemical inhibitors of calpain suppressed transglutamination. Our findings suggest that calpain contributes to the control of TGM1-mediated transglutamination and proteins cross-linked by transglutamination mask epitopes of TGM1.

## Introduction

The function of the skin as a barrier against the environment depends on the cornification of epidermal keratinocytes, which are then integrated into the stratum corneum at the surface of the skin. Cornification is a special mode of cell death that is characterized by massive cross-linking of proteins, which converts a living keratinocyte into a metabolically inactive cell corpse that is resistant to mechanical, chemical and microbial stressors^1,2^. This unique cell death-associated protein cross-linking is catalyzed by transglutaminases (TGMs), which establish isopeptide bonds between glutamine and lysine residues of different proteins^3,4^.

TGM1 is anchored through covalently linked palmitic acid in the plasma membrane of differentiated keratinocytes where it initiates epidermal cornification^5,6^. Cross-linking of periplakin, envoplakin, loricrin, small proline-rich proteins (SPRRs) and other proteins in close proximity to the plasma membrane leads to the formation of the so-called “cornified envelope”^4^. Later, omega-hydroxyceramides are covalently attached on the outside of the cornified envelope. A possible role of TGM1 in the attachment of ceramides^7^ has not been confirmed yet^8^. Inactivating mutations of human *TGM1* and targeted deletion of *Tgm1* in the mouse lead to aberrant cornified envelopes and stratum corneum defects. The human TGM1 mutations cause lamellar ichthyosis^9–11^, whereas loss of *Tgm1* in mice is lethal within few hours after birth^12^. Besides *TGM1*, *TGM3* and *TGM5* are expressed in human epidermis where they contribute to cornification. However, mutations of *TGM3* and *TGM5* cause only relatively mild epidermal phenotypes^13–16^.

TGM1 is regulated at the level of gene transcription and protein function. The expression of *TGM1* is upregulated during keratinocyte differentiation and reaches its peak in the granular layer of the epidermis. The TGM1 protein is, however, catalytically inactive until the cytoplasmic concentration of calcium ions (Ca^2+^) exceeds 20 $\upmu$M^17^. This cytoplasmic Ca^2+^ concentration increases by an unknown mechanism, possibly involving specific calcium channels, in cells of the granular layer just prior to their cornification^18–20^. In addition to the dependence on an elevated Ca^2+^ concentration, partial proteolysis of TGM1 has been suggested to contribute to the full activation of TGM1^21–24^. Specifically, cleavage of full-length TGM1 at two sites was reported to generate an N-terminal fragment containing the membrane anchor, a central fragment containing the catalytic triad, and a C-terminal fragment that remains complexed with the central fragment^23^. However, the significance of proteolysis for the enhancement of TGM1 activity has been controversial^25^ and the identity of the TGM1-processing protease, with cathepsin D and calpain being considered candidates, has not been fully clarified^21,26,27^. Likewise, the relevance of phosphorylation on TGM1 activity has remained uncertain^28^.

Various methods of detecting TGM activity in tissues and cells have been developed. The principle of these assays is the exposure of TGM enzymes to suitable substrates, such as Q/K-containing peptides or cadaverine, that are linked with a fluorophore, in the presence of high concentrations of Ca^2+^ ions^29,30^. In some studies, TGM activity patterns were compared with the in situ distribution of TGM proteins on serial sections of tissues^29^. To the best of our knowledge, there have, however, been no studies aimed at testing whether the levels of TGM proteins and TGM activity always correlate or display types of uncoupling indicative of TGM activation or inactivation.

Here, we show that TGM1 immunolabeling and TGM activity signals do not overlap in late differentiation of epidermal keratinocytes and in cells transfected with TGM1. In vitro assays suggest that the differential changes of TGM1 immunoreactivity and catalytic activity are linked to calpain, indicating a yet-uncharacterized regulation of epidermal cornification.

## Results

### Double labeling of human skin indicates persistent transglutaminase activity despite loss of immunoreactivity of TGM1 prior to epidermal cornification

**Figure 1:**
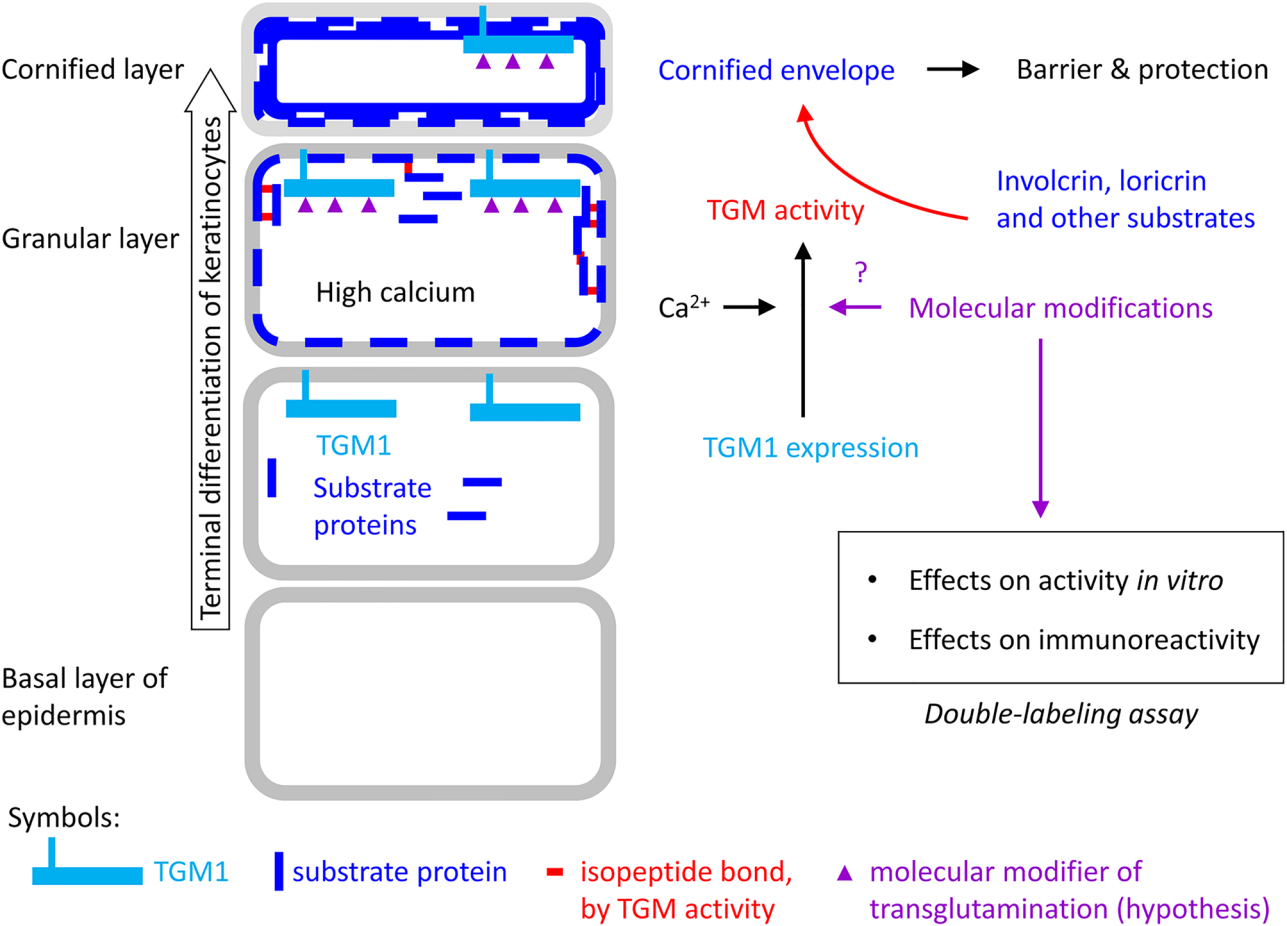
Control of TGM1 during keratinocyte differentiation. Keratinocytes differentiate as they move towards the skin surface. TGM1 is expressed during late differentiation and requires high calcium concentrations for catalytic activity which leads to the cross-linking of substrate proteins in the granular layer of the epidermis. The present study was designed to detect effects of molecular modifications on the in vitro activity and immunoreactivity of TGM1.

We hypothesized that, in addition to the elevation of the cytoplasmic calcium concentration, molecular modifications of TGM1 or its substrates affect the activity of TGM1 in keratinocytes during epidermal cornification and that such molecular modifications would also affect the activity and possibly the immunoreactivity of TGM1 in vitro (Fig. 1). To test this hypothesis, we established a double-labeling assay in which tissue sections are incubated with a fluorophore-tagged transglutaminase substrate at an optimal calcium concentration and a pH that preferentially supports the activity of TGM1 relative to other TGMs^30,31^, followed by immunofluorescence labeling using a primary antibody against TGM1 (Supplementary Fig. S1).

**Figure 2:**
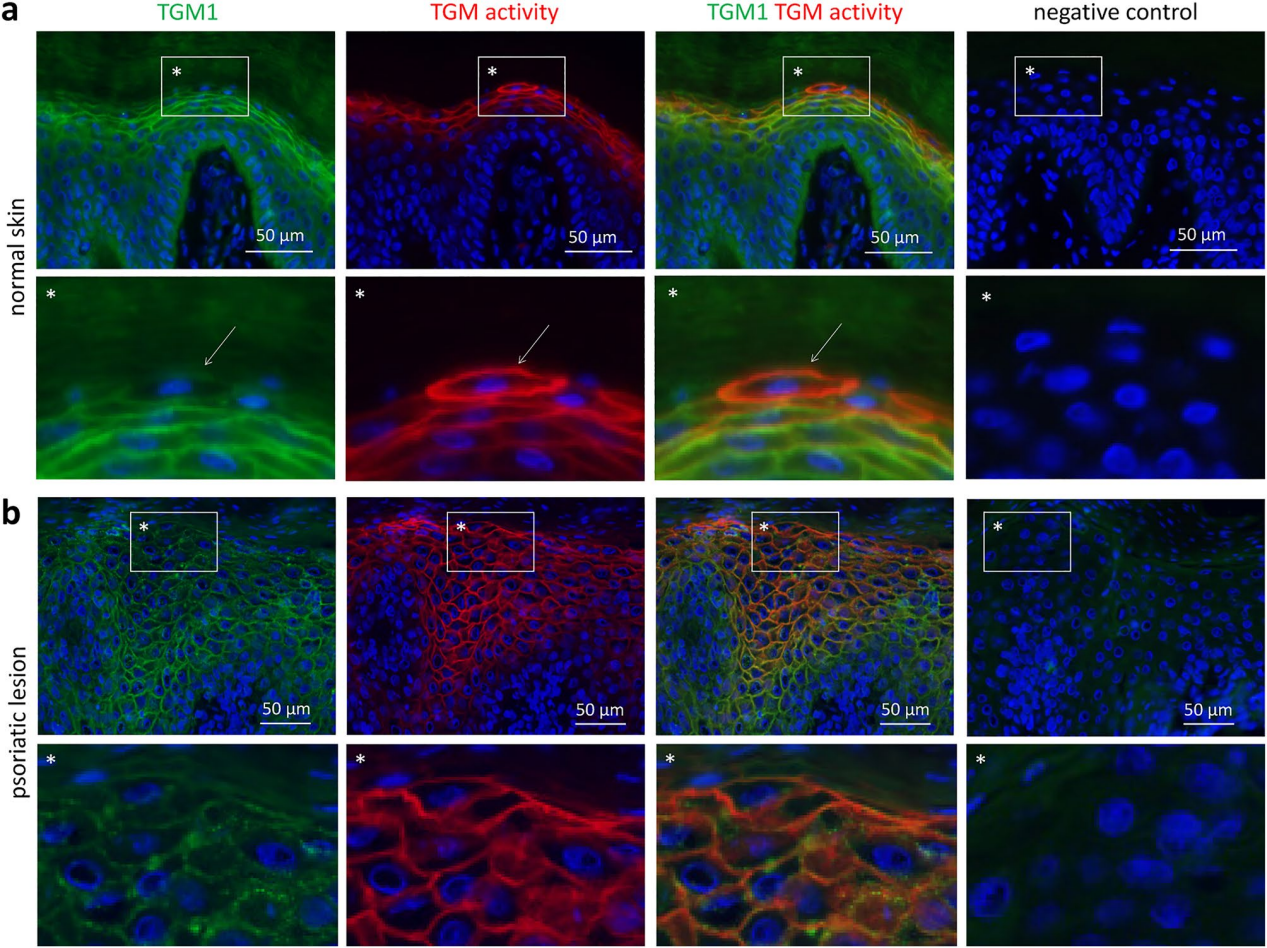
Immunodetection for TGM1 is reduced whereas transglutaminase activity persists in terminally differentiated keratinocytes in human epidermis. TGM activity and TGM1 immunofluorescence double-labeling was performed on sections of normal human skin (**a**) and lesional skin from patients with psoriasis (**b**). A white frame marks a detail of the image that is shown below at higher magnification (*). Note that normal skin (n = 3) and psoriatic skin (n = 5) were sampled in independent studies involving different body sites and storage of samples. Consequently, normal and psoriatic skin were investigated separately, and comparative analyses of healthy versus psoriatic skin were not performed. White arrows indicate cells of the uppermost layer of the living epidermis in which the TGM1 immunofluorescence signal is diminished or largely reduced, whereas TGM activity labeling is strong.

TGM1 protein, marked by immunolabeling, and TGM activity were detected in the granular layer of human epidermis (Fig. 2a). Both TGM1 immunolabeling and activity localized to the plasma membrane, corresponding to the expected membrane anchorage via the palmitoyl group at TGM1. In the outermost layer of nucleated cells, the immunolabeling signal of TGM1 decreased whereas the activity labeling remained prominent. In some cells of this layer (Fig. 2a, arrow), essentially only membrane-associated TGM activity was detectable without binding of the TGM1 antibody to these cells.

In psoriatic lesions, where TGM1 expression is known to be present at elevated levels^30^, the TGM1 immunosignal increased in lower layers of the epidermis and decreased again in the upper layers, whereas TGM activity was high up to the last living cell layer of the epidermis (Fig. 2b). Neither TGM1 immunolabeling nor activity labeling yielded signals in the parakeratotic (nuclear DNA-containing) cornified layer of psoriatic lesions. Negative control experiments, in which anti-TGM1 was replaced with immunoglobulin from non-immunized rabbits and the TGM activity labeling was blocked by omission of calcium and addition of divalent cation blocker EDTA, showed no signals in normal and psoriatic skin (Fig. 2).

### TGM1 immunolabeling and activity labeling in cultured cells shows that catalytically active TGM1 is only transiently immunodetectable

**Figure 3:**
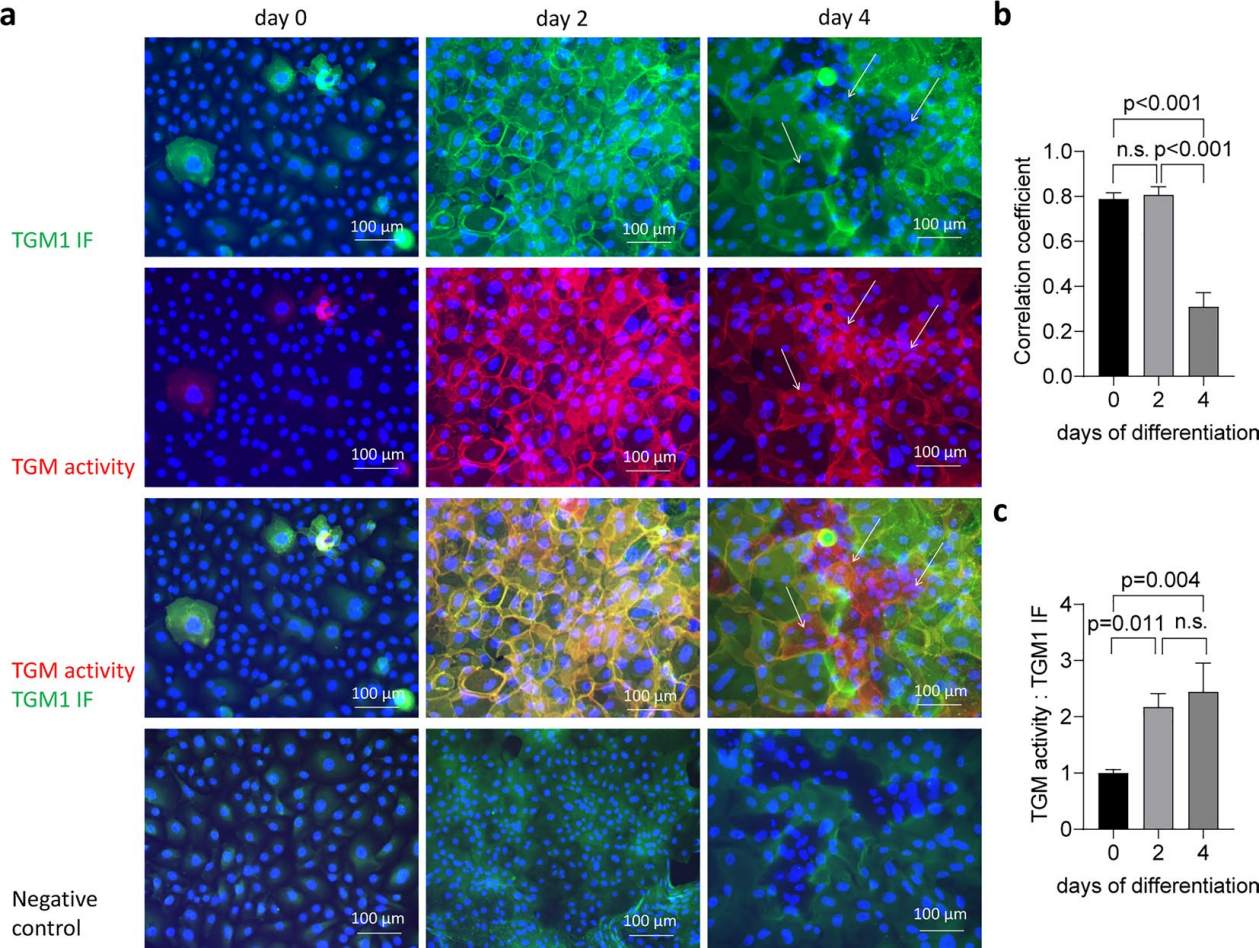
Transglutaminase activity persists after loss of TGM1 immunoreactivity in keratinocytes differentiating in vitro. Primary human keratinocytes were induced to differentiate by allowing them to reach 100% confluence (day 0) and subsequently culturing them in SKDM medium. (**a**) On days 0, 2 and 4 of differentiation, the cells were subjected to TGM activity labeling (red) and TGM1 immunofluorescence labeling (green). Nuclear DNA was labeled with Hoechst 33258 dye (blue). White arrows indicate clusters of cells displaying strong TGM activity and low TGM1 immunofluorescence. Three biological replicates were performed with similar results. (**b**) Pearson´s r correlation coefficient of TGM activity and TGM1 immunolabeling. Three images were used for the calculation of the correlation coefficient for each day. (**c**) The ratio of TGM activity and TGM1 immunofluorescence signals, normalized to the mean ratio at the day 0, is shown for each differentiation time point. Statistical significance was calculated using ordinary one-way ANOVA with p < 0.05 being considered significant. Bars and error bars represent mean values and standard deviations, respectively.

Next, we performed the TGM1 immunolabeling and activity double-labeling assay on human keratinocytes differentiating in vitro. Primary human epidermal keratinocytes were maintained in confluent culture, which triggers differentiation^32^. Initially, TGM activity was strictly colocalized with immunolabeling, but in later differentiation TGM1 immunolabeling was lost and TGM activity persisted (Fig. 3a). To quantify the relationship between TGM1 immunoreactivity and TGM activity during keratinocyte differentiation, we performed a colocalization analysis using Pearson´s correlation coefficient (r) as a measure of dependence between two image channels. Consistent with previous findings, Pearson´s r value was close to 1 (0.79 and 0.81, respectively) on days 0 and 2, and it decreased to 0.31 on day 4 (Fig. 3b), indicating that TGM activity and TGM1 immunoreactivity colocalize significantly less in a late stage compared to earlier stages of differentiation. Moreover, we analyzed the ratio of the TGM activity (red channel) and TGM1 immunoreactivity (green channel) intensity signals (Fig. 3c). This ratio increased significantly from day 0 (cells reaching confluence) to post-confluent states, corresponding to advanced differentiation, thus replicating the relative increase of TGM activity observed towards cornification in vivo (Fig. 2). The loss of TGM1 immunofluorescence despite persistence of TGM activity was in line with the hypothesis that TGM1 is modified in differentiated keratinocytes. However, these data did not allow to conclude that TGM1 remained active after loss of immunoreactivity because activity signals might derive from other TGMs expressed in keratinocytes.

**Figure 4:**
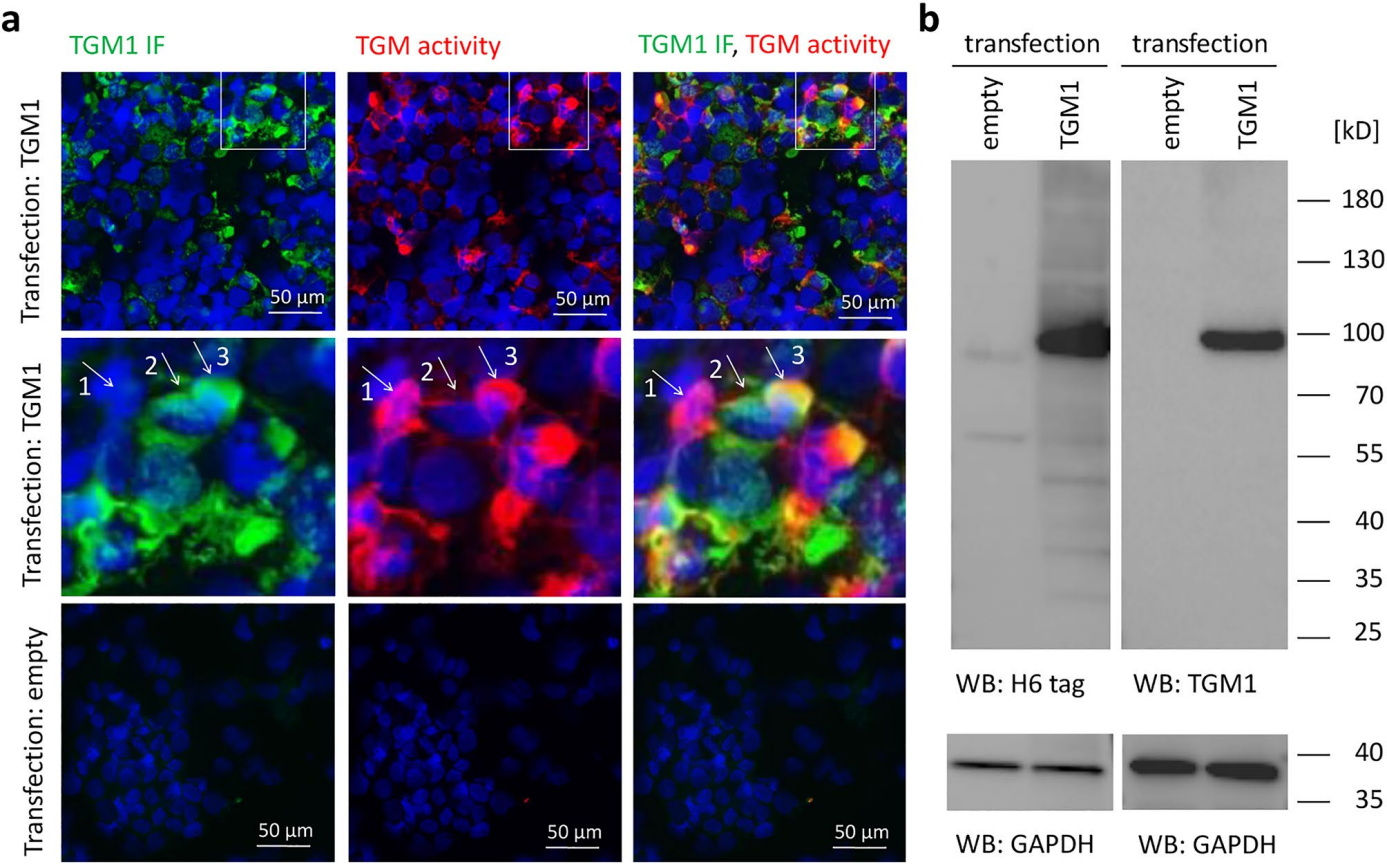
Transglutaminase activity persists after loss of TGM1 immunoreactivity in HEK293T cells. (**a**) HEK293T cells were transfected with TGM1, tagged with H6, or empty vector. The cells were collected by cytospin, fixed with methanol and subjected to TGM activity labeling (red) and immunofluorescence labeling with a primary antibody against TGM1 (green). Nuclear DNA was labeled with Hoechst 33258 dye (blue). White rectangles in images of the upper panels mark details that are enlarged in the middle panels. The arrows and numbers mark a cell exhibiting only TGM activity (arrow 1), a cell exhibiting only TGM1 immunofluorescence (arrow 2), and a cell displaying both TGM activity and TGM1 immunofluorescence (arrow 3). (**b**) Western blot analysis of cells transfected with empty vector or the expression vector for His-tagged (H6) TGM1. Primary antibodies against H6 and TGM1 were used to detect the recombinant protein with a predicted molecular mass of 100 kDa. GAPDH was analyzed as a loading control. Uncropped images of the membranes are provided in Supplementary Fig. S2.

To test whether TGM1 alone is able to yield the labeling pattern described above, that is, cells double-positive for TGM1 immunoreactivity and TGM activity and cells positive only for TGM activity, we transfected human embryonic kidney 293 (HEK293T) cells with recombinant TGM1 which contained an amino-terminal histidine 6 (H6) tag. HEK293T cells do not contain endogenous TGM activities, as demonstrated by the absence of TGM activity labeling signals in untransfected cells and in cells transfected with an empty vector (Fig. 4a, bottom panels). By Western blot analysis, TGM1 expression was detected in HEK293T cells only after transfection with TGM1 (Fig. 4b, Supplementary Fig. S2). Ectopic expression of TGM1 led to the appearance of TGM1 immunofluorescence and activity signals (Fig. 4a, upper panels). Of note, these TGM-derived signals were evenly distributed within cells, suggesting that in HEK293T cells ZDHHC13-dependent palmitoylation of TGM1^5^ did not efficiently target TGM1 to the plasma membrane in these cells. Importantly, the relative intensities of immunofluorescence and activity signals differed among transfected cells. Only a fraction of cells, exemplified by cell number 3 in Fig. 4a, displayed similar intensities of TGM1 immunolabeling and activity. Other cells, exemplified by cell number 2 in Fig. 4a, contained TGM1 and little, if any, TGM activity. Yet other cells, exemplified by cell number 1 in Fig. 4a, displayed TGM activity without being labeled with the anti-TGM1 antibody. Thus, the expression of TGM1 yielded TGM activity-positive cells in which TGM1 could not be bound by the anti-TGM1 antibody under the conditions of our assay. A similar immunofluorescence pattern has been obtained when an antibody against the His-tag was used instead of the anti-TGM1 antibody for immunolabeling of the H6-tagged TGM1 in HEK293T cells (Supplementary Fig. S3).

### Calpain small subunit 1 (CAPNS1) is an interaction partner of TGM1 in HEK293T cells

**Figure 5:**
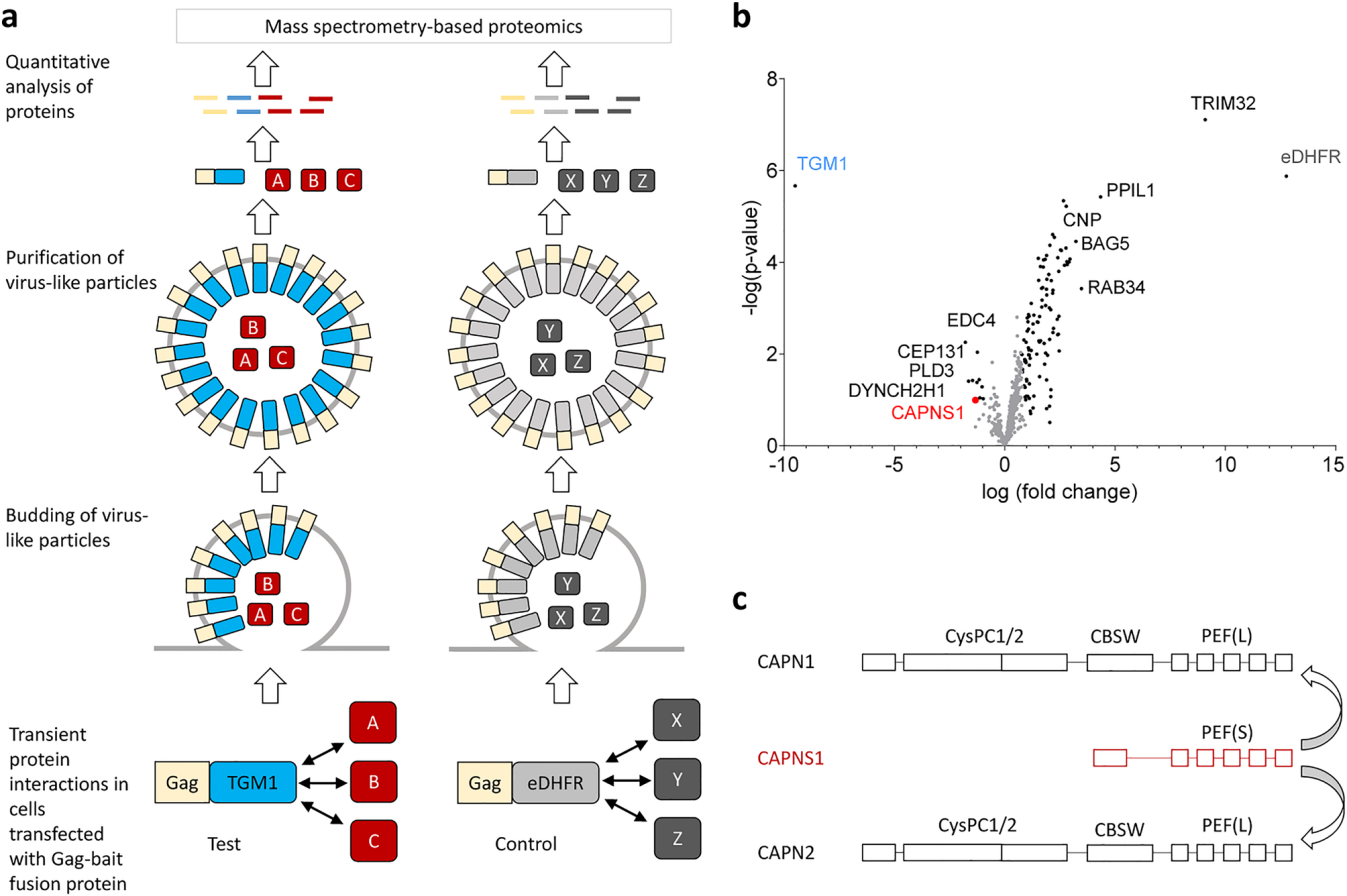
A Virotrap-based assay identifies calpain small subunit 1 (CAPNS1) as a possible TGM1 interactor. (**a**) Virotrap principle. A protein consisting of HIV-1 p55 Gag protein fused to the bait (TGM1, eDHFR as a negative control) is transiently expressed in HEK293T cells. Gag directs this fusion protein to the cell membrane and induces budding of virus-like particles (VLPs) in which interaction partners of the bait protein are trapped. The released VLPs are purified and the proteins are identified by mass spectrometry. (**b**) Volcano plot showing TGM1 interactors identified by Virotrap-based screening. TGM1-specific interaction partners are shown on the left side of the volcano plot and eDHFR-specific interaction partners are shown on the right side of the volcano plot. (**c**) The scheme depicts CAPNS1 interactions with calpain 1 (CAPN1) and calpain 2 (CAPN2) through the fifth penta-EF-hand (PEF) domain. eDHFR, *Escherichia coli* dihydrofolate reductase; CysPC1/2, cysteine protease core domain 1/2; CBSW, calpain-type β-sandwich.

To search for protein interaction partners of TGM1, we performed a Virotrap screening as schematically depicted in Fig. 5a^33^. In brief, Virotrap is based on the binding of proteins to a bait which itself is fused to the group-specific antigen (Gag) protein of HIV1. The interactors of this bait protein are trapped, together with the bait-Gag fusion protein, in virus-like particles, which bud from the producing cell. The virus-like particles are isolated and subsequently subjected to mass spectrometry-based proteomics. Using human TGM1 as a bait and *Escherichia coli* dihydrofolate reductase (eDFHR) as an unrelated control bait, we identified a small number of proteins enriched in TGM1-containing virus-like particles (Fig. 5b). Among them, calpain small subunit 1 (CAPNS1) stood out as a potentially relevant protein (Fig. 5b). CAPNS1 forms heterodimers with the calcium-activated cysteine proteases calpain 1 and calpain 2^34^ (Fig. 5c), which have previously been implicated in proteolytic processing of TGM1^27^, though it has remained unknown whether this proteolytic processing was important in the overall cornification process.

### Calpain inhibitors reduce TGM activity in differentiating human keratinocytes and transfected HEK293T cells

**Figure 6:**
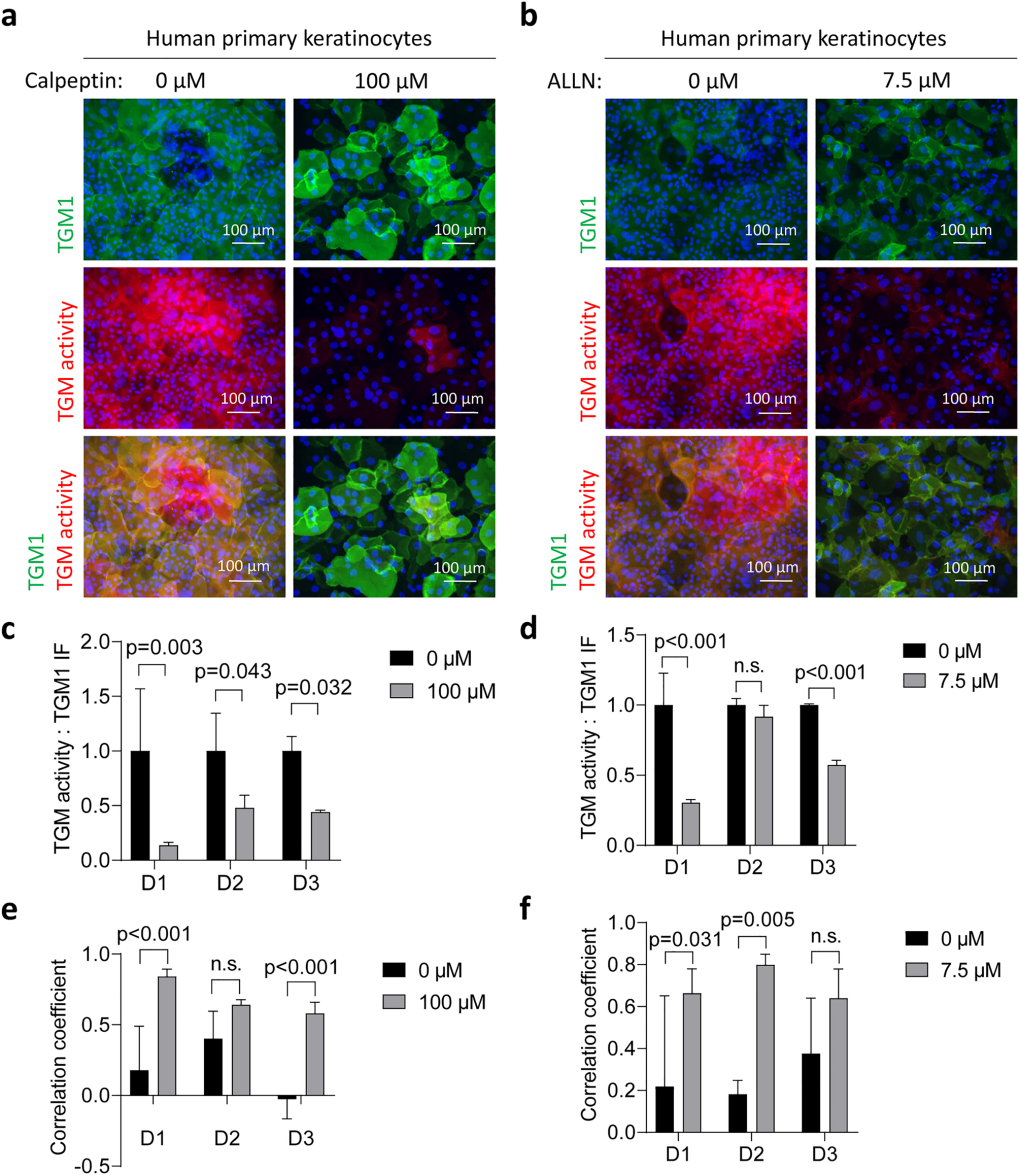
Calpain inhibitors block TGM activity and TGM1 and TGM activity signal separation. Human keratinocytes were differentiated for 4 days in the presence of calpeptin (**a**, **c**, **e**) or ALLN (**b**, **d**, **f**). Vehicle only (0 µM inhibitor) was used as control. (**a**, **b**) The cells were grown on chamber slides, fixed with methanol and subjected to TGM activity labeling (red) and TGM1 immunofluorescence labeling (green). (**c**, **d**) Ratio of TGM activity to TGM1 immunolabeling signals. The values of untreated cells were used for normalization. (**e**, **f**) Pearson´s r correlation coefficient of TGM activity and TGM1 immunolabeling. Note that treatment with calpain inhibitors enhanced the correlation between the residual TGM activity and TGM1 immunofluorescence due to the reduction of activity labeling in cells without TGM1 immunofluorescence. The significance of differences was calculated using unpaired t-test for n = 3 images for each donor (D1-D3) separately, with p < 0.05 being considered significant. Bars and error bars represent mean values and standard deviations, respectively.

To test whether the catalytic activity of calpain alters the immunoreactivity and/or activity of TGM1, we induced differentiation of primary human keratinocytes in vitro in the absence or presence of calpain inhibitors, ALLN and calpeptin. The cells were analyzed on day 4, the time point when TGM1 immunoreactivity declined in previous experiments (Fig. 3). The inhibitors were used at concentrations that allowed cellular metabolic activity, as determined by the CellTiter-Blue assay, of 84 ± 12% and 52 ± 10% of vehicle-treated cells in two independent experiments of ALLN treatment and 91 ± 18% and 84 ± 26% (mean ± SD) of vehicle-treated cells in two independent experiments of calpeptin treatment (Supplementary Fig. S4). In differentiating keratinocytes, calpeptin and ALLN led to a reduction of TGM activity, while detection of TGM1 was not impaired (Fig. 6a,b). The ratio of TGM activity versus TGM1 immunofluorescence upon treatment was reduced in cells from all three donors investigated (Fig. 6c,d). Moreover, colocalization analysis using Pearson´s r correlation coefficient indicated that inhibition of calpain led to strong colocalization of the residual TGM activity with TGM1 immunoreactivity (Fig. 6e,f), which corresponds to suppression of TGM activity-positive and TGM immunofluorescence-negative cells, as observed in the absence of calpain inhibitors. These findings indicate that inhibition of calpain reduces TGM activity, defined here as the linkage of the cadaverine-fluorophore conjugate to cellular proteins, and the loss of TGM1 immunoreactivity.

**Figure 7:**
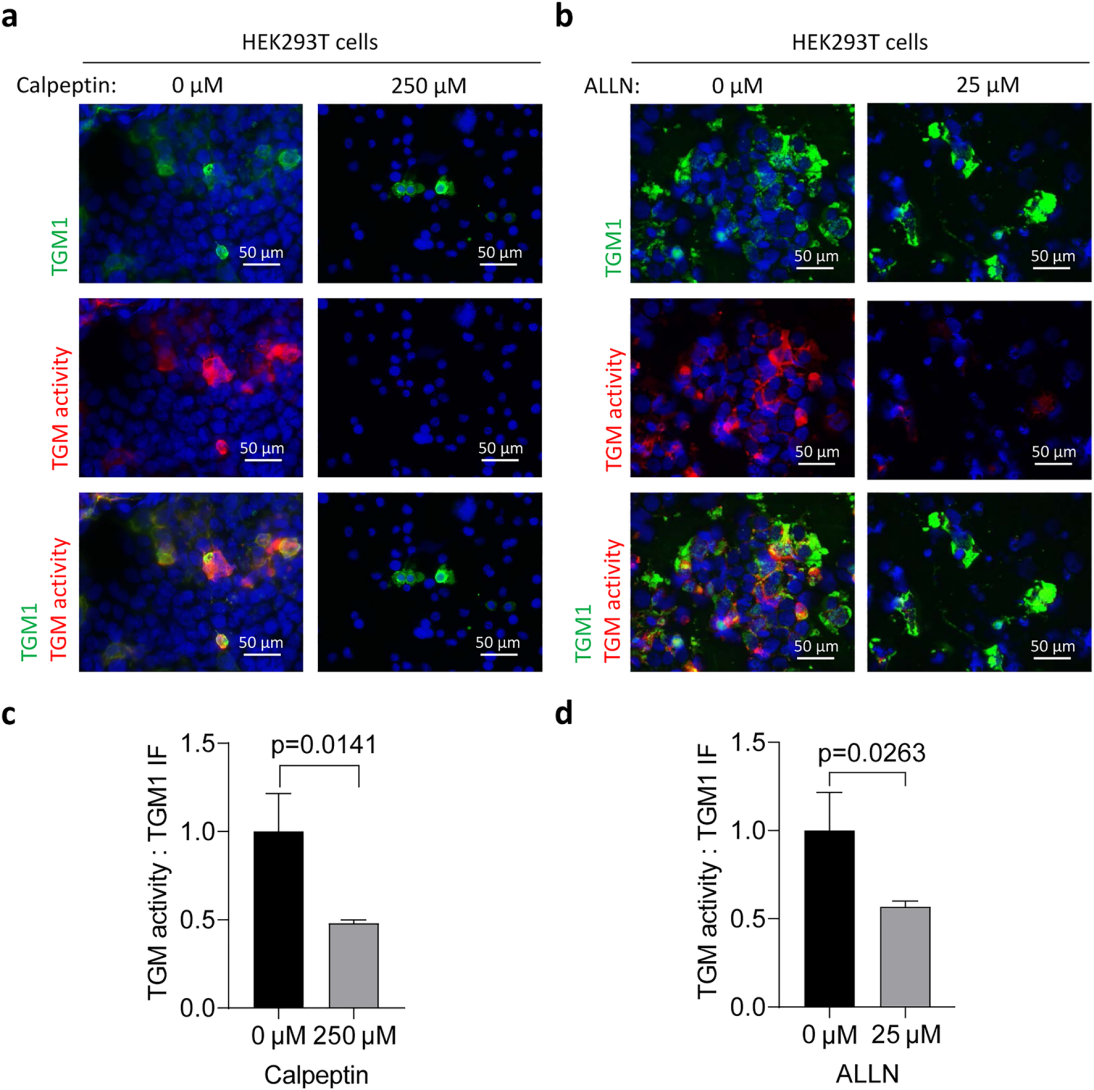
Calpain inhibitors block TGM1 activity in HEK293T cells. HEK293T cells were transfected with TGM1 followed by 48 h treatment with calpeptin (**a**, **c**) or ALLN (**b**, **d**). (**a**, **b**) The cells were collected by cytospin, fixed with methanol and subjected to TGM activity labeling (red) and TGM1 immunofluorescence labeling (green). (**c**, **d**) Ratio of TGM activity to TGM1 immunolabeling signals. The values of untreated cells were used for normalization. The significance of differences was calculated using unpaired t-test, with p < 0.05 being considered significant. Number of replicates, n = 3. Bars and error bars represent mean values and standard deviations, respectively.

To investigate the effects of calpain inhibition on TGM1 alone, the chemical inhibitors ALLN and calpeptin were tested on TGM1-transfected HEK293T cells. The cells were kept in the transfection medium for 6 h, after which the medium was changed and the inhibitors were added for 48 h. Treatment with the inhibitors slightly reduced TGM1 expression in HEK293T cells. However, it was evident that in the cells expressing TGM1 at appreciable levels, TGM activity was largely blocked (Fig. 7a,b) and the ratio of TGM activity versus TGM1 immunoreactivity was significantly reduced (Fig. 7c,d). Importantly, ALLN and calpeptin reduced TGM activity only when applied during the culture of cells and not when applied to the TGM1-positive cells before the TGM activity assay, indicating that they did not directly inhibit TGM1 (Supplementary Fig. S5). This pattern suggested that the calpain inhibitors suppressed an as-yet-undefined process required for the TGM1-mediated transglutamination reaction in cultured cells.

### Pretreatment of tissue sections with proteases allows the immunodetection of TGM1 in cornified keratinocytes

**Figure 8:**
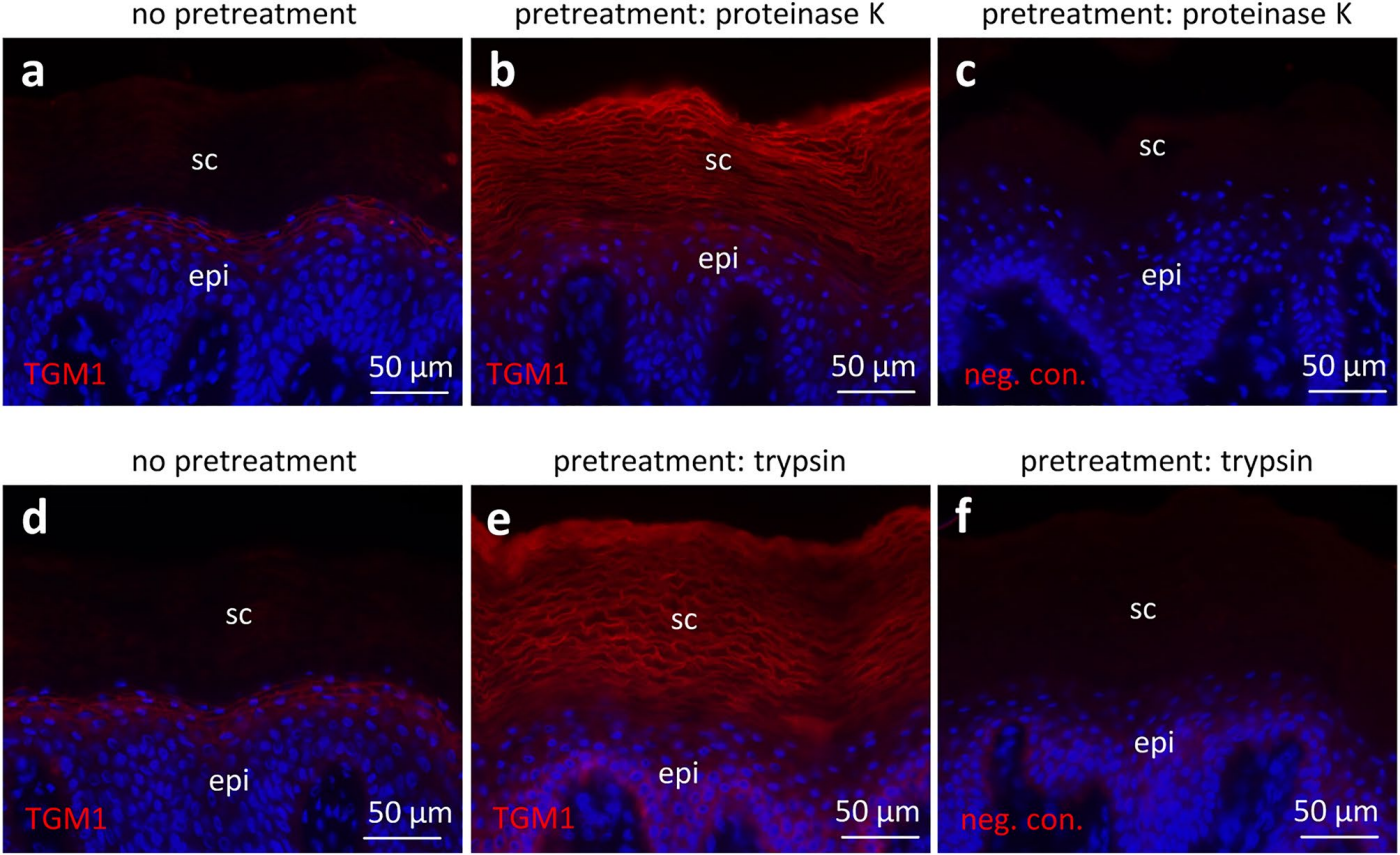
Protease pretreatment of skin sections facilitates immunolabeling of TGM1 in cornified cells. Human toe skin was immuolabeled for TGM1 without (**a**, **d**) or with pretreatment of the section with proteinase K (**b**) or trypsin (**e**). In negative control experiments (**e**, **f**), the sections were pretreated and the anti-TGM1 antibody was replaced by immunoglobulin G from non-immunized animals. epi, epidermis; sc, stratum corneum.

The absence of TGM1 immunolabelling in the latest stages of keratinocyte differentiation in vivo (Fig. 2) and in vitro under conditions permitting TGM activity (Figs. 3 and 6) suggested that anti-TGM1 antibodies could not bind their epitopes during and after cornification. We hypothesized that proteins cross-linked by TGM activity mask the epitopes of TGM1, similar to the masking of loricrin epitopes in cornified keratinocytes^35^. Therefore, we treated skin sections with proteinase K or trypsin prior to immunolabeling. Both pretreatments allowed the subsequent immunolabeling of TGM1 not only in entire granular layer but also in the stratum corneum of human skin (Fig. 8). The restoration of TGM1 immunoreactivity by partial degradation of proteins suggests that TGM1 is enclosed by other proteins which prevent the access of antibodies in the native state of the stratum corneum.

## Discussion

**Figure 9:**
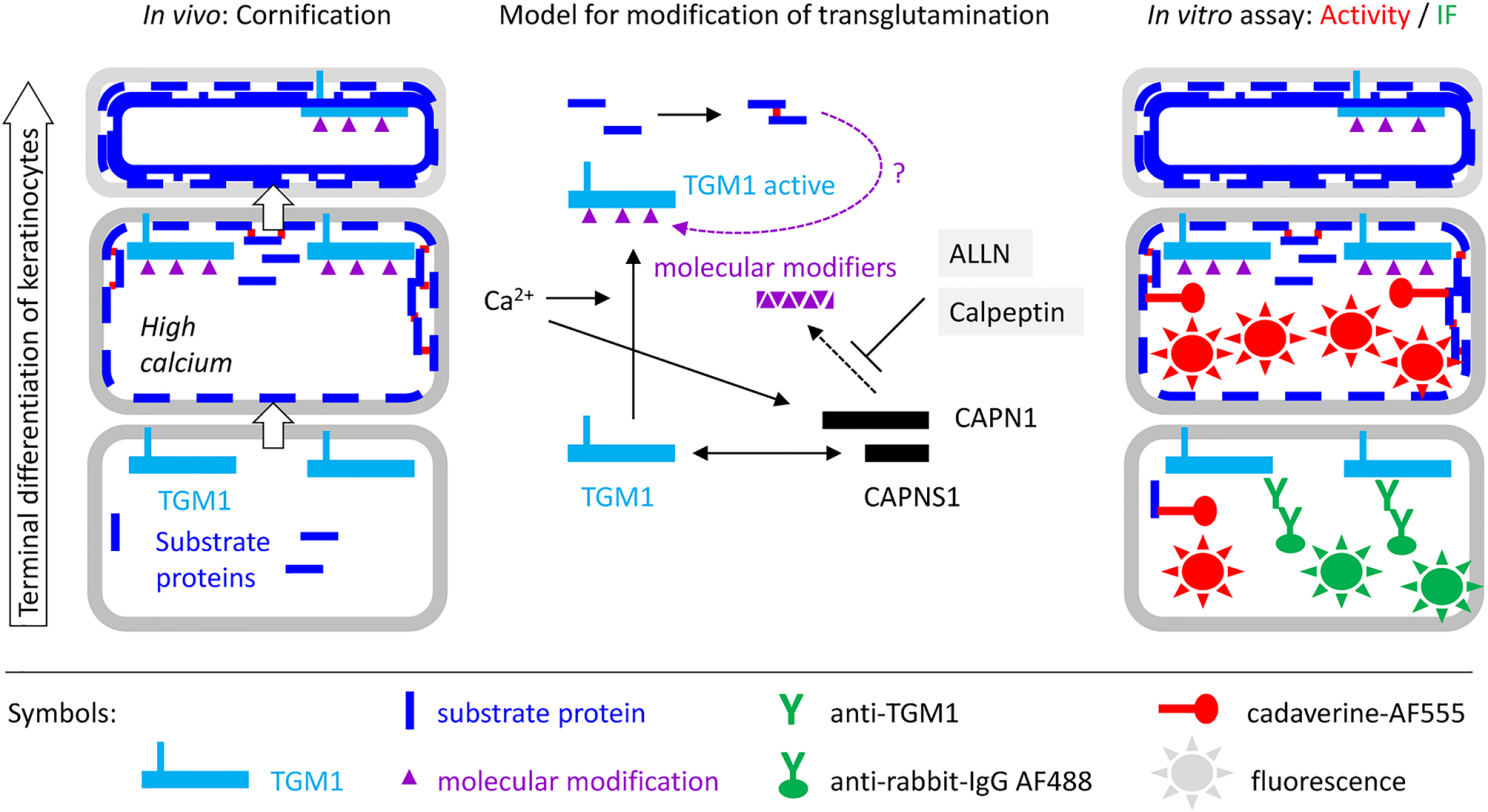
Model for modification of transglutamination during terminal differentiation of keratinocytes. The schematic model is based on the known dynamics of protein crosslinking during epidermal differentiation, which depends on a high calcium concentation immediately before cornification, and on the results of this study. A TGM activity and TGM1 immunofluorescence (IF) double labeling assay under optimal calcium concentration revealed colocalization of activity (red) and immunofluorescence (green) signals in incompletely differentiated cells, whereas TGM activity and no immunofluorescence signals were detected in terminally differentiated keratinocytes. CAPNS1 was identified as interaction partner of TGM1, and the calpain inhibitors, ALLN and calpeptin, blocked activity but not immunodection of TGM1. Together, these data suggest that calpain alters transglutamination of substrate proteins in a way that decreases the affinity or accessibility of TGM1 to anti-TGM1 antibodies and thereby prevents immunodetection of TGM1 in cornifying keratinocytes. As the molecular modifiers of TGM1 immunoreactivity are sensitive to proteolysis, TGM1-mediated cross-linking of proteins or peptides is a possible mechanism of masking TGM1 epitopes (indicated by a discontinuous arrow and a question mark).

TGM1 is a crucial enzyme for protein cross-linking in the course of epidermal cornification^10,12^. Here, we established an in vitro assay that allows parallel detection of TGM1 protein and TGM activity. The assay was primarily intended to determine changes of TGM activity that are not caused by changes of the expression level of TGM1. Surprisingly, our results suggest that some cells, especially in the most differentiated living layers of the epidermis, contain catalytic TGM activity despite the absence of a TGM1 immunofluorescence signal. This study was limited to the use of only one polyclonal TGM1 antibody and did not investigate whether the loss of immunoreactivity was confined to the part of TGM1 that is bound by the antibody used here. However, using the same antibody and pretreatment of skin sections with either proteinase K or trypsin (Fig. 8), we show that the TGM1 is retained even in the cornified layer of the epidermis. Furthermore, our experiments with TGM1-transfected HEK293T cells indicate that active TGM1 can lose its immunoreactivity in our TGM1 double labeling assay (Fig. 4). The immunoreactivity of the H6 tag of the recombinant TGM1 protein is decreased in a similar way in transfected cells that display high TGM activity (Suppl. Fig. S3). Thus, our results suggest that a modification of TGM1-expressing cells blocks immunolabeling of TGM1 (Fig. 9). In human skin, this modification is achieved by proteins or protein fragments which can be sufficiently removed by pretreatment of tissue sections with proteases. Both cells in the granular layer of the epidermis and cultured cells that express TGM1, undergo massive changes of their proteome because of TGM activity, suggesting that the activity of TGM1 itself may lead to the loss of TGM1 immunodetection under the conditions of our assay.

TGM1 is expressed in several suprabasal layers of the epidermis, but its catalytic activity is required mainly in the uppermost granular layer, when keratinocytes initiate cornification. Therefore, to prevent premature protein cross-linking, TGM1 activity has to be tightly regulated. In vitro labeling of TGM activity on tissue sections has demonstrated that the catalytic activity of TGM1 is absent in lamellar ichthyosis^24,29,36^ and levels of active TGM1 are increased in psoriasis^30^. The combination of TGM activity labeling with immunofluorescence labeling of TGM1, as used in the present study, may help to further characterize conditions with aberrant activation or processing of TGM1. Our investigation of psoriatic lesions suggests that, like in normal skin, TGM activity labeling is stronger than immunolabeling in the uppermost epidermal layers, suggesting that the cornification-associated decline of relative TGM1 immunoreactivity is at least partially maintained in these lesions. However, it is important to note that the optimal conditions applied on tissue sections (Supplementary Fig. S1), including uniformly high calcium ion concentrations during TGM activity labeling, may differ substantially from conditions in diseased skin.

The TGM1 immunolabeling and TGM activity in situ labeling assay may serve as a tool to characterize the natural activation of TGM1 and the effects of chemical compounds on TGM1 in cultured cells. Our double labeling assay determines the relation of TGM1 protein antigenicity (binding by TGM1 antibody) and TGM activity. The TGM activity assay was not strictly TGM1-specific although the pH conditions favored TGM1-mediated activity over the activity of other TGMs^30,31^. Specific detection of TGM1 activity may be achieved by using fluorescent peptide K5 as substrate^37^. The null hypothesis was that the immunolabeling and the activity labeling, performed at optimal conditions on the entire sample, would perfectly co-localize. Intriguingly, we found that in differentiated keratinocytes and TGM1-transfected HEK293T cells, TGM1 immunoreactivity is only transiently detectable and lost when TGM activity is still present. These data obtained in cell culture suggested that cultured cells could be used to screen for interaction partners potentially involved in the processing of TGM1.

Virotrap-based screening for TGM1 interaction partners identified CAPNS1 as a candidate protein (Fig. 5b), which suggested a link between calpain and TGM1. We did not test a possible role of CAPNS1 alone, but investigated the roles of the catalytically active complexes of calpains 1 and 2, in which CAPNS1 functions as a chaperone for the catalytic subunits CAPN1 and CAPN2, respectively. Interestingly, the calpain inhibitor, ALLN, has already been reported to affect TGM activity through blocking calpain-mediated activation of TGM1^27^. Moreover, recombinant TGM1 was shown to be cleaved by calpain 1 and calpain 2 in vitro^25^. Calpain 1, like TGM1, is expressed in the granular layer of the epidermis^38,39^. However, proteolysis of TGM1 by calpain 1 was reported to leave the catalytic activity of purified recombinant TGM1, as measured in a microtiter plate assay, unaffected^25^. In the present study, treatment of keratinocytes and transfected HEK293T cells with two calpain inhibitors, ALLN and calpeptin, suppressed TGM1 activity while TGM1 remained immunodetectable. While ALLN inhibits also other cysteine proteases, such as cathepsins L and B, calpeptin is monospecific for calpains. In contrast to a published study using a different anti-TGM1 antibody^25^, we did not detect western blot bands indicative of direct calpain-mediated cleavage of TGM1. Thus, our data support the hypothesis that calpains 1 and/or 2 are involved in the enhancement of TGM1-mediated transglutamination, but the mechanism of this control is presently unknown. Of note, calpain cleaves filaggrin, filaggrin 2 and hornerin^38–41^, which are cross-linked to the cornified envelope of differentiated keratinocytes. It is conceivable that calpain-dependent fragments of these and other proteins are preferred substrates of transglutaminases and allow more efficient cross-linking than the corresponding full-length proteins.

In three experimental settings (sections of human epidermis, human keratinocytes differentiating in vitro and TGM1-transfected HEK293T cells), immunolabeling of TGM1 was lost in a fraction of cells, corresponding to the most differentiated cells in keratinocytes, which contained TGM activity. Theoretically, this loss of immunolabeling may occur through one or more of the three following mechanisms: 1. Degradation of TGM1 and presence of activity by other TGMs; 2. Covalent modification of TGM1 that inhibits binding of the anti-TGM1 antibody to its epitopes; 3. Blockade of access to TGM1 epitopes via other molecules.

Degradation of TGM1 is a possible cause of reduced immunofluorescence labeling in the upper epidermis where TGM activity might be derived not only from TGM1 but also from TGM3 and TGM5^42^. However, TGM1 can be detected by mass spectrometry-based proteomic analysis of human stratum corneum^43,44^. Importantly, degradation of TGM1 cannot explain loss of TGM1 immunosignals in TGM1-transfected HEK293T cells displaying TGM activity, because the entire TGM activity derives from ectopically expressed TGM1 in these cells. Assuming that the degradation-independent mechanism of loss of TGM1 immunolabeling is not specific for HEK293T cells, it appears reasonable to predict that this mechanism also operates in epidermal keratinocytes. Consequently, degradation of TGM1 is not considered the key contributor to the signal patterns in the TGM1 immunofluorescence and activity labeling, although degradation of a fraction of TGM1 may occur during or after cornification of keratinocytes.

The second possible cause of suppressed immunolabeling is the introduction of one or more post-translational modifications, such as proteolytic cleavage, glycosylation, phosphorylation, deimination or ubiquitination to TGM1. These and other covalent modifications can disrupt epitopes of antibodies. Lack of immunoreactivity depends on the type of the modification of the antigen and on the precise epitopes of the antibodies. We performed western blot analysis of TGM1 and did not detect bands corresponding to fragments of TGM1 (Fig. 4b), which could be due to the genuine absence of fragments or due to lack of binding of the anti-TGM1 antibody to TGM1 fragments. In any case, there is no experimental support for the logical candidate of a calpain-dependent modification of TGM1, namely proteolytic cleavage. Instead of fragments smaller than full length TGM1, we detected a slight smear above the predicted TGM1 band when an antibody against the His-tag of recombinantly expressed TGM1 was used, whereas no high-molecular mass signals were detected with the anti-TGM1 antibody. In this regard, one could speculate that the molecular mass of TGM1 may be increased by cross-linking with other proteins or peptides, catalyzed by active TGM1 itself. Intriguingly, covalent linkages of peptides to TGM2 have been detected in independent studies^45,46^. To the best of our knowledge, cross-linking of peptides to TGM1 has not been reported yet.

The third possible mechanism of loss of TGM1 immunodetection in differentiated keratinocytes is “masking of epitopes”, which means, the prevention of antigen–antibody binding through other molecules. In the case of TGM1, it is plausible to assume that TGM1-mediated cross-linking of proteins and peptides generates a high-molecular weight polypeptide structure, an intermediate to the forming cornified envelope, in its close proximity. TGM1 and many other antigens are not detected in fully cornified keratinocytes of the stratum corneum, suggesting that cornification itself leads to the blockade of epitopes. The cornification-associated masking of loricrin epitopes was demonstrated previously^35^, and the unmasking of TGM1 epitopes by treatment of tissue sections by proteinase K or trypsin (Fig. 8) strongly suggests that proteins or protein fragments prevent binding of the anti-TGM1 antibody. In contrast to antibodies, the cadaverine-fluorophore conjugate appears to reach active TGM1 in such a situation, thereby generating the TGM activity signal that is observed in late differentiated keratinocytes. Likewise, peptides generated by calpain or other proteases, may be available as substrates of TGM1 even when the cornified envelope has started to form and the contact of TGM1 to more bulky proteins is sterically hindered. This hypothesis remains to be tested in future studies.

## Materials and methods

### Ethics statement

Human tissue samples of healthy donors used for histological investigations and for the isolation of primary keratinocytes were obtained from skin excised in plastic surgery procedures. Informed consent was obtained from all participants. The study was conducted according to the guidelines of the Declaration of Helsinki and approved by the Ethics Committee of the Medical University of Vienna (Votes 601/2009, 217/2010, and 1,285/2013; approval EK. 1969/2021, date of approval November 18, 2021). Further skin samples were obtained from a study that was conducted according to the guidelines of the Declaration of Helsinki and approved by the Ethics Committee of the Medical University of Vienna (approval EK. Nr. 131/2004, date of approval 19 April 2004)^47^. Samples from psoriatic lesions were collected in a clinical study that was conducted according to the guidelines of the Declaration of Helsinki and approved by the Ethics Committee of the Medical University of Vienna (approval number 1408/2022, date of approval 31 May 2022).

### Cell culture and transfection

Primary human keratinocytes were isolated from skin excised in plastic surgery procedures (n = 3) as described before^48^. Keratinocytes were grown in Keratinocyte Growth Medium 2 (KGM2, PromoCell GmbH, Heidelberg, Germany). After the keratinocytes reached 100% confluence, differentiation was induced by replacing the medium with serum-free keratinocyte-defined medium (SKDM), consisting of KGM2 without bovine pituitary extract and epinephrine, and supplemented with 1.2 mM CaCl_2_ (Sigma Aldrich), 50 µg/mL of L-ascorbic acid (catalogue number A4544, Sigma Aldrich) and 0.1% fatty acid-free bovine serum albumin (BSA) (catalogue number K35-002, PAA Laboratories). For the time-course experiments, the cells were prepared for the assay on a respective day of differentiation. For the calpain inhibition experiments, ALLN calpain inhibitor 1 (catalogue number A6185, Sigma-Aldrich) or calpeptin (catalogue number 03-34-0051, Merck) were added to the SKDM medium at a final concentration of 7.5 µM and 100 µM, respectively. Cells were allowed to differentiate in the presence of the inhibitor for 4 days. For the control experiments, an equivalent volume of the vehicle, dimethyl sulfoxide (DMSO) was added. The medium was replaced with fresh medium every second day, with or without inhibitors corresponding to the experimental condition.

HEK293T cells were maintained in DMEM medium (catalogue number 41965–039, Gibco) supplemented with 10% FBS (catalogue number 10091148, Gibco), 1 mM sodium pyruvate (catalogue number 11360070, Gibco), 2 mM L-glutamine (catalogue number 25030081, Gibco) and 100 U/ml penicillin–streptomycin (catalogue number 15140122, Gibco). For the transfection, 0.5*10^6^ cells were plated per well in a 6-well plate. 24 h after plating, transfection was performed using Lipofectamine 2000 (catalogue number 11668019, ThermoFisher) according to the manufacturer´s protocol. In brief, 5 µg of the plasmid DNA and 7.5 µl of Lipofectamine 2000 were diluted in 250 µl of Opti-MEM and added dropwise onto the cells kept in 1.75 ml of antibiotics-free DMEM. The plasmid consisting of N-terminally H6-tagged TGM1 cloned into a pcDNA3.4 vector was purchased from GenScript (New Jersey, USA). The cells were incubated in the transfection medium for 6 h, after which the medium was replaced by fresh DMEM and the corresponding inhibitors were added.

## CellTiter-Blue assay

The CellTiter-Blue assay (catalogue number G8080, Promega), which measures the ability of living cells to convert resazurin into fluorescent resorufin, was performed according to the manufacturer´s protocol on primary human keratinocytes differentiated for 4 days with or without calpain inhibitor as described above. The fluorescent signal was measured using a Promega plate reader using emission filter 580–640 nm and green excitation filter at 520 nm.

### *Histology, transglutaminase *in situ* activity assay and immunofluorescence analysis*

Fresh tissue samples were dissected and washed in phosphate-buffered saline (PBS). The samples were placed in cryomolds (Tissue-Tek® Cryomold®, Sakura FineTec, USA) filled with optimal cutting temperature (OCT) compound (Scigen, Paramount, Canada) and snap-frozen in liquid nitrogen. Cryo-sections of 6 µm thickness were prepared with a cryostat (Leica CM3050S) and stored at −20 °C or stained immediately. Primary human keratinocytes grown on chambered cell culture slides (catalogue number 354118, Falcon, NY, USA) and a diluted cell suspension of transfected HEK293T cells prepared by cytospin were fixed on the slides using 100% methanol at −20 °C for 30 min, air dried and stored at −20 °C or stained immediately. An in situ transglutaminase activity assay combined with immunofluorescence was performed based on published protocols^30,49–51^. Cryosections of human skin were thawed at room temperature for 10 min before they were placed in PBS for 5 min at room temperature to remove remaining OCT compound from the slides. The samples were encircled with a liquid blocker (Daido Sangyo Co. Ltd. Tokyo, Japan) and subsequently incubated with 2% BSA in PBS with 0.05% Tween at room temperature for 30 min. Subsequently, the samples were incubated with 5 µM Alexa Fluor 555 cadaverine (catalogue number A30677, Thermo Fisher Scientific, Waltham, MA, USA) in 0.1 M Tris–HCl pH 7.4 with either 5 mM CaCl_2_ to facilitate transglutaminase activity or 5 mM EDTA to suppress transglutaminase activity (negative control) for 2 h at room temperature under protection from light. The reaction was stopped by incubating the samples in 25 mM EDTA in PBS for 5 min after which the samples were rinsed in PBS. The samples were then incubated overnight at 4 °C with TGM1 antibody (rabbit polyclonal antibody against full length human TGM1, catalogue number A018, Zedira, Darmstadt, Germany)^52^ diluted to the final concentration of 6.25 µg/ml in 2% BSA in PBS with 0.05% Tween. The sections were subsequently washed with PBS 3 times for 5 min, followed by incubation with donkey anti-rabbit immunoglobulin antibodies conjugated to Alexa-Fluor 488 (AF488) (Molecular Probes) and 1 µg/ml nuclear label Hoechst 33258 (Molecular Probes) for 30 min at room temperature. Afterwards, the sections were rinsed in PBS and mounted with Permafluor (catalogue number TA-030-FM, Thermo Fisher Scientific, Waltham, MA, USA). Sections were studied with an Olympus BX63 light microscope, and images were taken with an Olympus UC-90 camera. Fluorescent images were taken and merged with cellSens Dimension software (version 1.16).

### Protease pretreatment of cryosections and immunofluorescence

Cryo-sections of 6 µm thickness were prepared from human toe samples with a cryostat (Leica CM3050S) and stored at −20 °C. Cryosections were thawed at room temperature for 10 min before they were placed in PBS for 5 min at room temperature to remove remaining OCT compound from the slides. For pretreatment with proteases, cryosections were incubated for 20 min at room temperature with 5 µg/mL proteinase K (catalogue number 3115879001, Roche, Mannheim, Germany) or 0.008% Trypsin–EDTA (catalogue number 25300054, Gibco). The sections were washed 2 times with 4% BSA in PBS followed by 90 min incubation with 4% BSA in PBS. The samples were then rinsed and incubated overnight at 4 °C with anti-TGM1 antibody (rabbit polyclonal antibody against full length human TGM1, catalogue number A018, Zedira, Darmstadt, Germany)^52^ diluted to the final concentration of 2.5 µg/ml in 4% BSA in PBS. The sections were subsequently washed with PBS 3 times for 5 min, followed by incubation with goat anti-rabbit immunoglobulin antibody conjugated to Alexa-Fluor 546 (AF546) (Molecular Probes) and 1 µg/ml nuclear label Hoechst 33,258 (Molecular Probes) for 30 min at room temperature. Afterwards the sections were rinsed in PBS and mounted with Permafluor (catalogue number TA-030-FM, Thermo Fisher Scientific, Waltham, MA, USA). Sections were studied with an Olympus BX63 light microscope, and images were taken with an Olympus UC-90 camera using cellSens Dimension software (version 1.16).

### Quantification and colocalization analysis of TGM activity and TGM1 immunofluorescence signals

The signal intensities of TGM activity and TGM1 immunofluorescence were measured using ImageJ software. Mean signal intensity of each channel was measured and normalized to the signal corresponding to the total number of cells. The ratio of normalized signals of TGM activity and TGM1 immunofluorescence was calculated for 3 random images per condition. The intensity ratio of each image was normalized to the mean intensity ratio of the corresponding control images. The images to be compared were always treated equally. Unpaired t-test was used to calculate significance between the treated and untreated groups. The Pearson´s r correlation coefficient was measured using JACoP colocalization ImageJ plug-in^53^. This analysis was performed on 3 random images per condition. An unpaired t-test was used to calculate the significance between the treated and untreated groups.

### Western blot analysis

HEK293T cells were lysed in a buffer containing 50 mM Tris–HCl (pH 7.4), 2% SDS and complete protease inhibitor cocktail (Roche, Mannheim, Germany) and homogenized by sonication. After removal of insoluble debris by centrifugation, 15 μg protein was electrophoresed through a sodium dodecyl sulfate (SDS) polyacrylamide gel (Bio-Rad) and thereafter blotted onto a nitrocellulose membrane. Mouse anti-H6 (catalogue number MA1-21315, Invitrogen)^54^ and rabbit anti-TGM1 antibodies (catalogue number A018, Zedira)^52^ were used as primary antibodies at a dilution of 1:500, followed by goat anti-rabbit immunoglobulin G (catalogue number 31463, Thermo Fisher) and sheep anti-mouse immunoglobulin G (catalogue number NXA931V, Amersham) conjugated to horseradish peroxidase as secondary antibodies at a dilution of 1:10,000. Membranes were re-probed with mouse anti-GAPDH antibody (catalogue number 5G4cc, HyTest, Turku, Finnland) at a dilultion of 1:5,000, using sheep anti-mouse immunoglobulin G (catalogue number NXA931V, Amersham) conjugated to horseradish peroxidase as secondary antibody at a dilution of 1:10,000. Enhanced chemiluminescence reagent (catalogue number 1859024, Thermo Fisher) was used to develop the signal.

### Virotrap analysis

Virotrap-based screening of TGM1 interaction partners was performed according to a published approach^33^. In brief, human TGM1 (GenBank accession number: NP_000350.1) was fused to the carboxy-terminus of p55 Gag, and the recombinant chimeric protein was used as bait. *Escherichia coli* dihydrofolate reductase (eDHFR) fused to Gag was used as control. HEK293T cells were transfected with bait-Gag fusion constructs together with expression constructs for vesicular stomatitis virus glycoprotein (VSV-G) and FLAG-tagged VSV-G^33^. FLAG-tagged VSV-G is integrated into VLPs and allows efficient antibody-based purification of VLPs. The Virotrap particles were lyzed and digested with trypsin, the resulting peptides were separated by nano-liquid chromatography and identified by mass spectrometry as described previously^33,55^. The mass spectrometry proteomics data have been deposited to the ProteomeXchange Consortium via the PRIDE^56^ partner repository.

## Data availability 

The mass spectrometry proteomics data have been deposited to the ProteomeXchange Consortium (http://proteomecentral.proteomexchange.org) via the PRIDE partner repository^56^ with the dataset identifier PXD045105.

### Supplementary Information

Supplementary Information.
