# Supplementary material for: Cornification of keratinocytes is associated with differential changes in the catalytic activity and the immunoreactivity of transglutaminase-1
Source: Sci Rep. 2023 Dec 6;13:21550. doi: 10.1038/s41598-023-48856-1 (PMC10700374; doi:10.1038/s41598-023-48856-1)
Supplement: Supplementary file 1 — Supplementary Information. [file 41598_2023_48856_MOESM1_ESM.pdf]

Supplementary Data: Supplementary Figures

Cornification of keratinocytes is associated with differential changes in the catalytic activity and the immunoreactivity of transglutaminase-1

Marta Surbek, Tessa Van de Steene, Attila Placido Sachslehner, Bahar Golabi, Johannes Griss, Sven Eyckerman, Kris Gevaert, Leopold Eckhart

Content

Supplementary Figures S1-S5

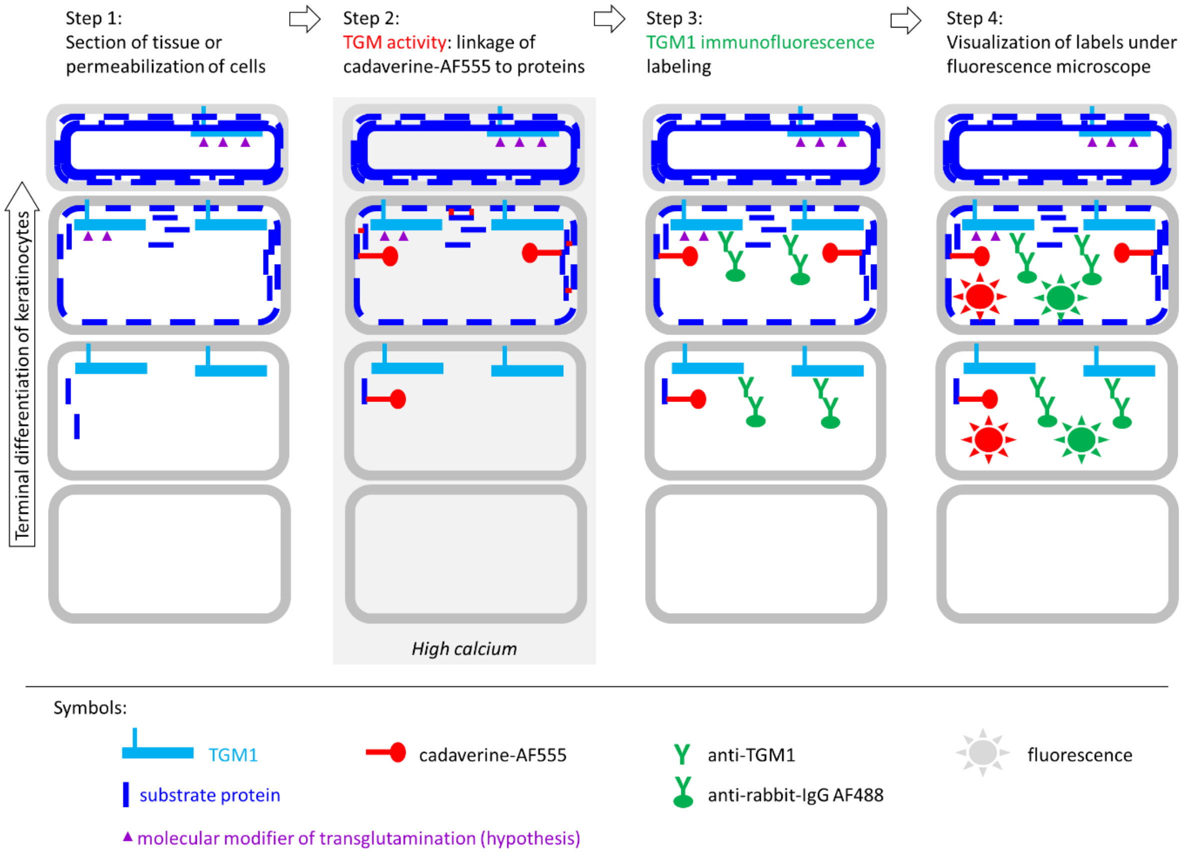

Suppl. Fig. S1. Schematic depiction of the key steps of the TGM1 immunolabeling and activity labeling assay. The assay is performed on a section of tissue or on fixed and permeabilized cells. In the first step, the slides are incubated with cadaverine-AF555 diluted in a high calcium containing buffer. This allows active transglutaminase to cross-link cadaverine-AF555 to proteins. Subsequently, the sections are incubated with an antibody binding to TGM1 protein, followed by second-step anti-rabbit-IgG conjugated with the fluorophore AF488. The fluorescent labels are visualized under a fluorescence microscope.

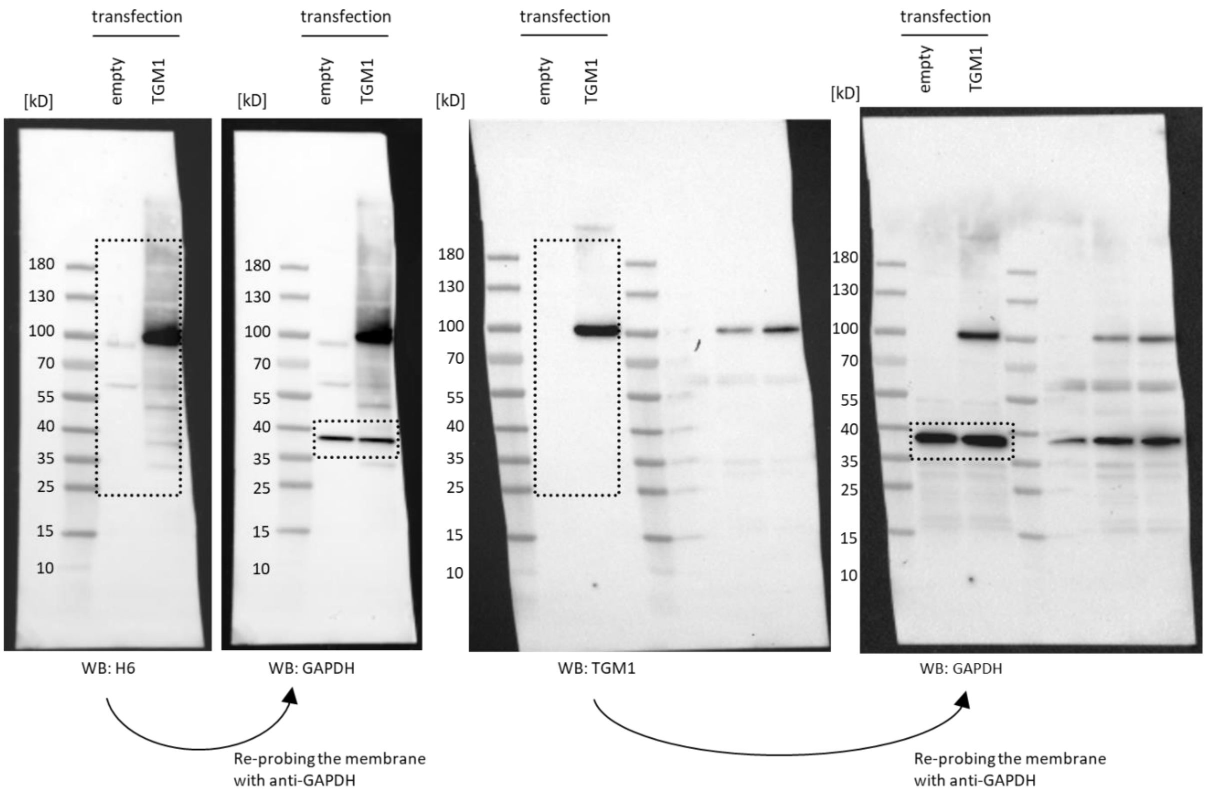

Suppl. Fig. S2. Uncropped images of the Western blots shown in Figure 4b. HEK293T cells were transfected with empty vector or the expression vector for His-tagged (H6) TGM1, and the lysates from the cells were subjected to Western blot analysis with primary antibodies against H6 and TGM1. The membranes were reprobed with anti-GAPDH as described in the "Materials and methods" section. The chemiluminescence signals are merged to the images of the membranes and the bands of the molecular weight marker (kD, kilo-Dalton). The images shown in Figure 4b are marked by dotted lines and the corresponing langes labelled on the top.

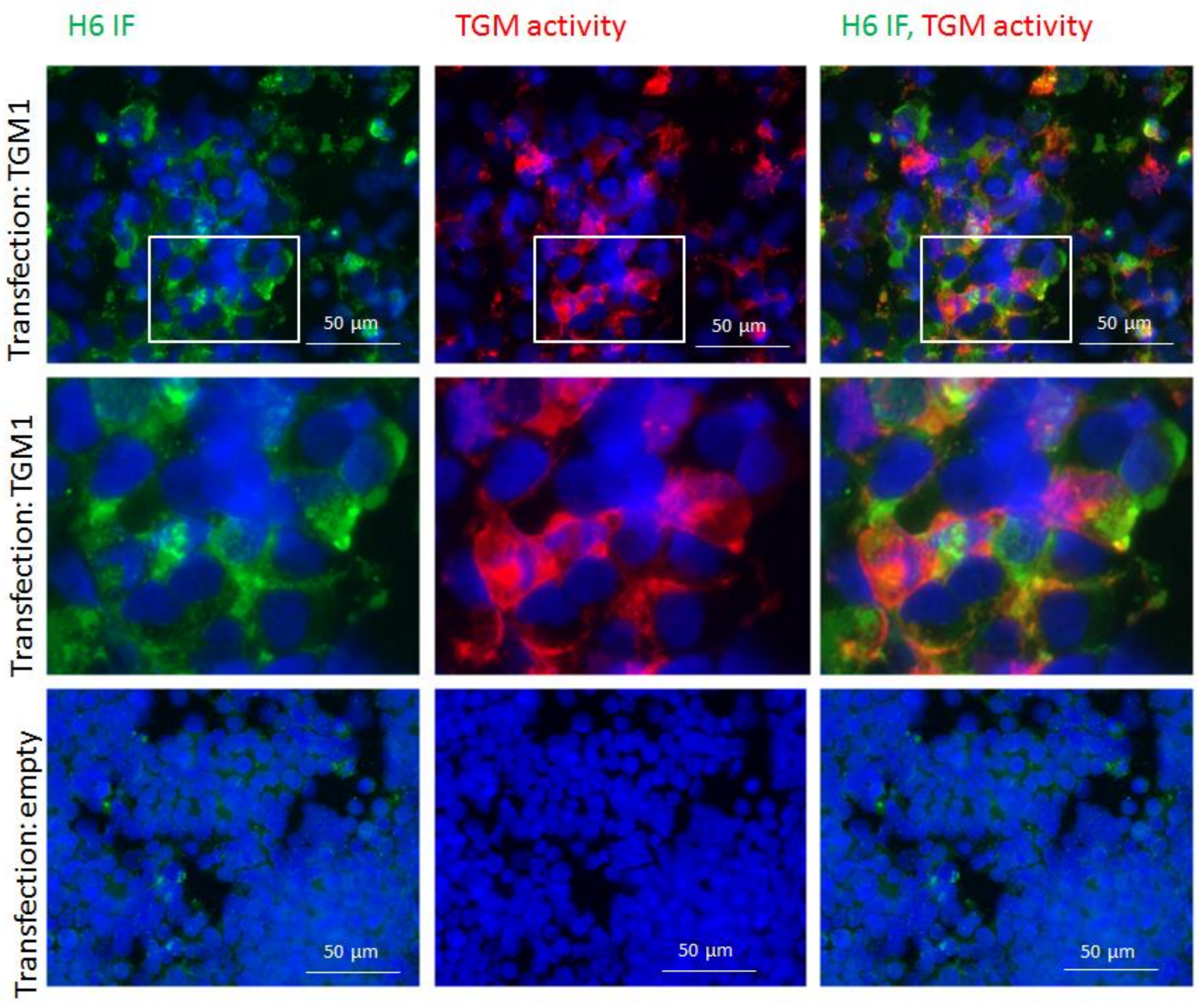

Suppl. Fig. S3. Immunolabeling of histidine 6 (H6) tag and transglutaminase (TGM) activity in H6-TGM1-transfected HEK293T cells. HEK293T were transfected with TGM1 containing an amino-terminal histidine 6 (H6) tag. The cells were double-labeled by immunofluorescence (IF) using anti-H6 antibody (green) and TGM activity labeling using Alexa Fluor 555 cadaverine (red). The second row of images shows details that are marked by white frames in the first row of images. Note that, similar to the results obtained the double-labeling experiments using anti-TGM1 antibody (Figure 4) instead of anti-H6, a fraction of cells display only TGM activity, while immunolabeling is reduced.

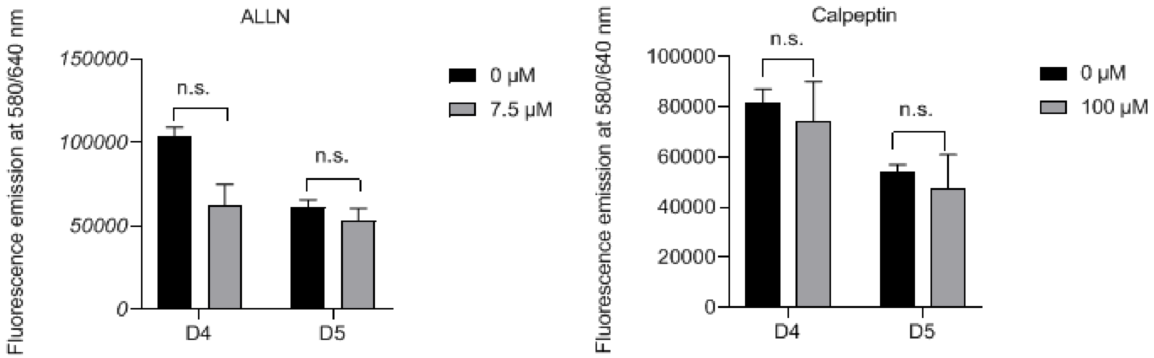

Suppl. Figure S4. Viability assay of ALLN and calpeptin-treated keratinocytes. Primary human keratinocytes from healthy donors (n=2) were allowed to differentiate in the presence or absence of the corresponding calpain inhibitors for 4 days. After 4 days, CellTiter-Blue cell viability assay was performed according to the manufacturer´s protocol. The intensity of fluorescence emission for n=3 replicates at 580/640 nm is showed in the graphs. Stastical significance was calculated using ratio paired t-test, with p-value < 0.05 considered significant. Bars represent mean value and standard deviation.

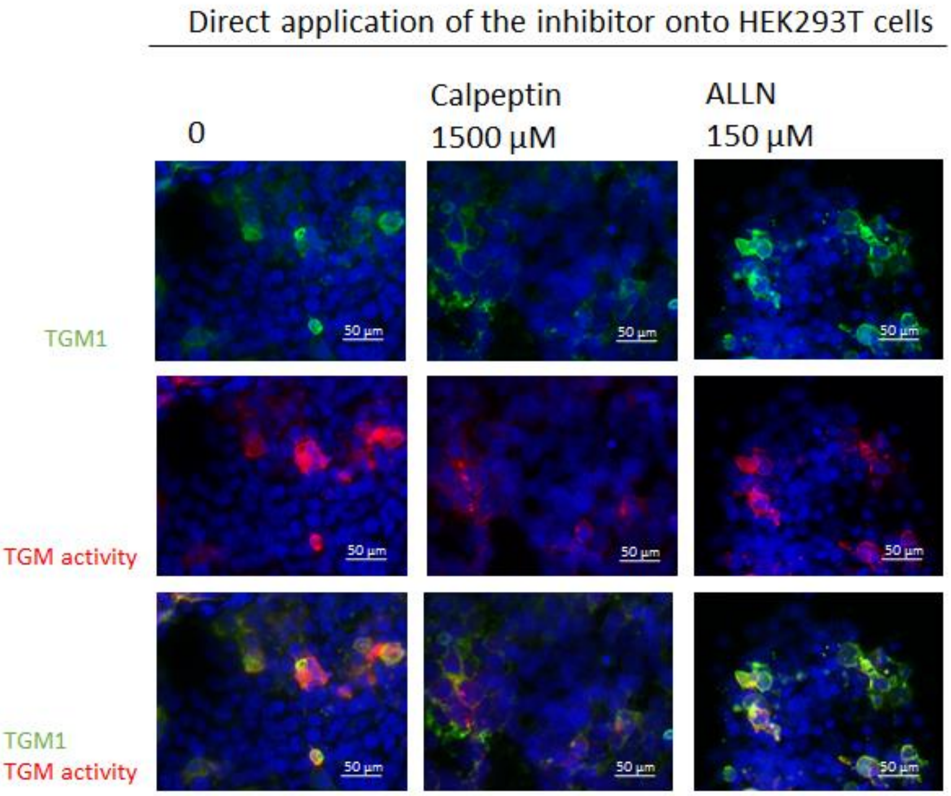

Suppl. Fig. S5. Calpeptin and ALLN do not directly inhibit TGM activity. TGM1-transfected HEK293T cells were prepared by cytospin. Prior to the TGM activity and TGM1 immunofluorescence double-labeling assay, the methanol-fixed cells were incubated with calpeptin or ALLN at concentrations corresponding to the 6-fold concentration used for the treatment of cultured cells (Figure 7). Application of calpain inhibitors prior to the assay without previous treatment of living cells did not suppress TGM activity labeling, indicating that these chemicals did not directly interfere with TGM activity.
